# TNF Signaling Dictates Myeloid and Non-Myeloid Cell Crosstalk to Execute MCMV-Induced Extrinsic Apoptosis

**DOI:** 10.3390/v12111221

**Published:** 2020-10-28

**Authors:** Pratyusha Mandal, A. Louise McCormick, Edward S. Mocarski

**Affiliations:** 1 Department of Microbiology and Immunology, Emory Vaccine Center, Emory University School of Medicine, Atlanta, GA 30322, USA; 2 BioMarin Pharmaceuticals, Novato, CA 94949, USA; alouisemccormick@gmail.com

**Keywords:** apoptosis, extrinsic apoptosis, intrinsic apoptosis, pyroptosis, necroptosis, inflammation, TNF, IFN, DNA virus, cytomegalovirus, replication, HCMV, MCMV, UL36, M36, vICA, Caspase-8, myeloid cells, cell death

## Abstract

Cytomegaloviruses all encode the viral inhibitor of caspase-8-induced apoptosis (vICA). After binding to this initiator caspase, vICA blocks caspase-8 proteolytic activity and ability to activate caspase-3 and/or caspase-7. In this manner, vICA has long been known to prevent apoptosis triggered via tumor necrosis factor (TNF) family death receptor-dependent extrinsic signaling. Here, we employ fully wild-type murine cytomegalovirus (MCMV) and vICA-deficient MCMV (∆M36) to investigate the contribution of TNF signaling to apoptosis during infection of different cell types. ∆M36 shows the expected ability to kill mouse splenic hematopoietic cells, bone marrow-derived macrophages (BMDM), and dendritic cells (BMDC). Antibody blockade or genetic elimination of TNF protects myeloid cells from death, and caspase-8 activation accompanies cell death. Interferons, necroptosis, and pyroptotic gasdermin D (GSDMD) do not contribute to myeloid cell death. Human and murine fibroblasts or murine endothelial cells (SVEC4-10) normally insensitive to TNF become sensitized to ∆M36-induced apoptosis when treated with TNF or TNF-containing BMDM-conditioned medium. We demonstrate that myeloid cells are the natural source of TNF that triggers apoptosis in either myeloid (autocrine) or non-myeloid cells (paracrine) during ∆M36 infection of mice. Caspase-8 suppression by vICA emerges as key to subverting innate immune elimination of a wide variety of infected cell types.

## 1. Introduction

Programmed cell death pathways provide well-orchestrated defense mechanisms leading to clearance of unnecessary, injured, or infected cells during development, disease, and pathogen invasion. Apoptosis is an evolutionarily conserved programmed cell death pathway, either mediated by external factors (extrinsic apoptosis) or by intracellular stress (intrinsic) induced by DNA damage, hypoxia, organelle dysfunction, infection, and metabolic inhibition [1]. Death receptors (DR) in the tumor necrosis factor (TNF) receptor superfamily engage with respective ligands such as TNF binding to the TNF receptor (TNFR)1 and FasL binding to Fas (CD95) to drive the formation of cytosolic signaling complex [2,3,4,5]. Components of this complex have been described in detail before by Mocarski et al. [5]. Briefly, activation of DR by binding to ligands employs cytosolic adaptor Fas-associated via death domain (FADD) to scaffold association of caspase (CASP)8, cellular FLICE inhibitory protein (cFLIP), receptor-interacting kinase (RIPK)1 and RIPK3, for a complex known as IIb or the ripoptosome. This complex promotes activation of CASP8 via autoproteolytic cleavage. Cleaved CASP8 promotes cleavage activation of CASP3 and/or CASP7 to execute apoptosis. In many settings, inhibition of CASP8 unleashes RIPK3-dependent necroptosis [6].

All herpesviruses encode specific inhibitors of cell death pathways to sidestep innate and adaptive immunity [5,7,8,9,10,11,12,13,14]. Cytomegaloviruses (CMVs) are ubiquitous pathogens that infect mammals in a species-specific manner and rely on multiple cell death suppressors to prevent the elimination of infected cells. Human (H)CMV causes severe disease in the newborn following transplacental transmission, where this virus is a common cause of pathogen-associated birth defects [15,16,17], as well as in immunosuppressed patients, where retinitis, encephalitis, colitis, and pneumonitis predominate [18,19,20,21,22]. Viral replication and inflammatory signaling contribute in poorly understood ways to CMV disease pathogenesis. Murine (M)CMV is a related betaherpesvirus that allows the elaboration of host–pathogen interactions, antiviral immune mechanisms, and disease processes. CMV-encoded gene products suppress apoptosis (both extrinsic and intrinsic), as well as necroptosis, to facilitate sustained viral replication in cells and host mammals [5,23,24,25,26,27,28,29]. The viral inhibitor of caspase 8-induced apoptosis (vICA) has been evolutionarily conserved in HCMV and MCMV [23,28], a unique feature compared to the dozens of other modulatory functions encoded by these diverse viruses. vICA-deficient MCMV or HCMV drives spontaneous CASP8-dependent apoptosis specifically in myeloid cells but not in fibroblasts [30,31,32,33]. Furthermore, vICA-deficient MCMV fails to disseminate efficiently in mice. Consistent with the expected mechanism of action [23,28], bone marrow-derived macrophages (BMDMs) from mice with deficiency in CASP8 and necroptotic kinase RIPK3 are not susceptible to murine vICA-deficient virus (∆M36)-induced apoptosis [33]. When infected, these mice exhibit normalized levels of dissemination that have been attributed to the inability of infected macrophages to support extrinsic apoptosis. Thus, vICA inhibition of CASP8 sustains replication fitness even though the connections between innate inflammation, CASP8-driven apoptosis, and viral replication in cells (both myeloid and non-myeloid) need greater substantiation. HCMV UL36-encoded vICA has recently been implicated in the stabilization of necroptosis mediator mixed lineage kinase domain-like (MLKL) in fibroblasts [34], in contrast to observations showing MCMV M36-encoded vICA sensitizes to necroptosis [33], a property common to other virus-encoded CASP8 inhibitors [35]. Understanding the full spectrum of contributions made by this viral protein to innate inflammatory signaling promises to resolve assay-dependent observations and reveal additional therapeutic approaches to control CMV disease. 

TNF-dependent signaling is intricately associated with CMV disease and infection. Fleck et al. [36] showed that apoptosis mediated by the TNF receptor (TNFR)1-dependent signaling and Fas contribute to control of viral infection as well as to patterns of inflammation in vivo. MCMV infection of BMDM or mice drives significantly elevated levels of TNF [33,37]. During MCMV-triggered encephalitis of neonatal mice or retinitis in adult mice, virus-induced TNF contributes to apoptosis and disease progression [38,39]. Inhibition of TNF-dependent signaling reduces tissue damage in these settings. Furthermore, viral immediate early gene expression has been intricately linked with TNF signaling during latency [40], although any role for immediate early gene UL36 or M36 [23] remains to be elucidated. Notably, HCMV commits UL138 function to induce TNFR1 levels on infected cells. Thus, the infected cell gains the advantages of elevated TNF signaling [41,42] while blocking CASP8 activation through the action of vICA [23,24,28,29,43]. When expressed independently of virus infection, vICA binds to the pro-domain of CASP8 [23] and prevents TNF-, Fas-, or RIPK3 inhibitor-dependent apoptosis in fibroblasts [30,44]. A similar mechanism is predicted to play out in infected cells. When Cicin-Sain et al. [31] followed up the initial observations of Menard et al. [29] seeking mechanisms that underly vICA function, FADD-DN was able to substitute for vICA and prevent macrophage apoptosis as well as support in vivo replication. At the time, TNFR1 (the primary death receptor mediating CASP8 activation) appeared dispensable for strain pSM3fr ∆M36 virus-induced dissemination. The behavior of pSM3fr and pSM3fr-derived mutants may be confounded by a spontaneous mutant disrupting expression of MCMV chemokine (MCK)2, proposed to be a mediator for efficient entry into myeloid cells [45]. Dissemination of MCMV strains uniformly depends on monocyte-derived macrophages [11,46,47,48,49,50], but MCK-2 contributes to entry only with the pSM3fr strain and not with the fully WT K181 strain [46,51]. K181 deploys MCK2 as a virus-encoded chemokine that recruits inflammatory [52] and patrolling monocytes [46] to modulate the host T cell response and enhance dissemination. Daley et al. [33] showed *Tnfa^−/−^Zbp1^−/−^* mice normalized K181-BAC-derived ∆M36 replication, suggesting a contribution of TNF signaling in the pathway suppressed by vICA. It is known that macrophage-derived TNF synergizes with IFNγ to limit pSM3fr bacmid-derived ∆M36 replication in murine embryonic fibroblasts (MEF; [32]). Given such complexities, experiments to determine how vICA interfaces with TNF-dependent antiviral pathways warrant further investigation with fully WT and paired ∆M36 mutant MCMV.

Here, we employ MCMV (K181) parental and vICA-deficient virus (∆M36) [33] to infect myeloid cells (the primary cell type responsible for virus dissemination in mammals) or other cell types (endothelial cells and fibroblasts) that support infection in vivo. BMDM, BM dendritic cells (BMDC), or hematopoietic cells from infected mice are all highly susceptible to this death. TNF blockade or gene elimination completely protects BMDM from apoptosis, revealing an autocrine role for this cytokine in macrophage apoptosis. vICA restrains death-associated inflammatory signaling in myeloid cells such that ∆M36 infection exhibited elevated levels of TNF production or processing of inflammatory cytokine IL-1β when compared to K181. ∆M36-induced death requires the presence of host CASP8; however, CASP8 is dispensable for TNF production from myeloid cells. Non-myeloid cells fail to produce TNF during infection. BMDM-derived supernatant or exogenous TNF induces death in ∆M36-infected endothelial cells or fibroblasts. Therefore, in all permissive cell types studied, vICA prevents TNF-dependent CASP8 activation and execution of apoptosis. Interestingly, human UL36 has long been known to restore vICA function during ∆M36 infection of cells or mice [28,53]. We show that ∆M36-infected human fibroblasts also synergized with TNF signaling for extrinsic apoptosis, supporting the concept that vICA functions similarly in both primate and murine betaherpesviruses. Overall, we demonstrate autocrine TNF-dependent signaling is required to observe ∆M36-induced, CASP8-dependent apoptosis in myeloid cells. In all CMV-infected cells, TNF signaling may eliminate infected cells unless CASP8 proteolytic activity is suppressed by vICA.

## 2. Materials and Methods

### 2.1. Cell Culture and Reagents

BMDM were generated as described previously [54]. Briefly, flushed marrows from tibias and femurs of 8- to 12-week-old mice were cultured for 7 days in the following medium: Dulbecco’s Modified Eagle Media (DMEM) containing 4.5 g/mL glucose (10-013 CV, Corning, Charlotte, NC, USA), 10% fetal bovine serum (F2442, Sigma-Aldrich, St. Louis, MO, USA) 2 mM l-glutamine (MT 25005CI, ThermoFisher Scientific, Marietta, GA, USA) supplemented with 100 units/mL penicillin and 100 units/mL streptomycin (MT 3002CI, Fisher). For BMDM culture, the medium had a final 20% fetal bovine serum and 20% filtered L929-conditioned medium (as a source of macrophage colony-stimulating factor). All BMDM experiments were performed within 9 days of the BM harvest. BMDC were generated as described previously [55]. Briefly, BM cells were cultured in complete medium supplemented with murine glanulocyte macrophage colony-stimulating factor (GM-CSF, 20 μg/mL, AF315-03, PeproTech, Canbury, NJ, USA) and murine interleukin-4 (IL-4, 5 ng/mL, AF-214-14, PeproTech) and used within 12 to 14 days with medium changes every 3 to 4 days. Only suspended cells were used for experiments. MEFs were collected from embryos <10 days old as described previously [56] and maintained in complete medium. All experiments with MEFs were performed within 5 passages of isolation. SVEC4-10 (ATCC CRL-2181), NIH-3T3, and foreskin-derived human fibroblasts (HFs) were maintained in complete medium and used within 10 passages. All cells were maintained at 37 °C in a humidified 5% CO_2_ incubator. zVAD-FMK (SM001) was from SM Biochemical, Anaheim, CA, USA; murine TNF (315-01A-20UG) and human TNF (300-01A) were from PeproTech, Cranbury, NJ, USA; and murine IFNβ (12401-1) and IFNγ (12500-2) were from PBL Assay Science, Piscataway, NJ, USA.

### 2.2. Virus and Mice

K181-BAC and K181-derived ∆M36 viruses have been described [33,57]. WT, as well as mutant (*Ifnar1^−/−^* and *Casp8^−/−^Ripk3^−/−^*) mice have been described [55] and were bred at Emory University under approved animal care protocol. For MCMV infection, 8- to 12-week-old mice were infected by intraperitoneal inoculation with 10^6^ PFU K181 or ∆M36 in 500 μL of complete medium. Mouse infections, the harvest of organs, and plaque assay of organ homogenates on NIH-3T3 were performed as described previously [33]. Equal volumes of splenic homogenates from each mouse were utilized for TNF (DY410, R&D Systems, Minneapolis, MN, USA) and IL-1β (DY 401, R&D Systems, Minneapolis, MN, USA) ELISAs. Recorded organ weight allowed determination of cytokine levels per gram of tissue. 

### 2.3. Viability, CASP8 Activity Assay, and ELISA

For cell viability or CASP8 activity assay, 10^4^ cells (for all cell types) were plated per well of 96-well plates (Corning, 3610). Twenty-four hours post-plating, cells were infected with the virus at multiplicity of infection (MOI) = 10. Virus incubation on cells for 1 h was followed by removal of inoculum and the addition of fresh complete medium. Drugs or cytokines were added after the inoculation of cells with virus (1 hpi) to avoid any interference with viral entry or initial steps in replication. ATP loss and CASP8 activity were assayed using Cell Titer-Glo (G9241, Promega Corporation, Madison, WI, USA) and Caspase-Glo 8 (G8200, Promega Corporation, Madison, WI, USA), respectively, from Promega. For ELISA or treatment of SVEC4-10 cells, cell supernatants were collected from infected cells at the indicated times post-infection. Supernatants were centrifuged at 1000× *g* for 5 min at 4 °C to remove cells or debris. Sterile filtered cell-free supernatant was added to SVEC4-10 cells or utilized for TNF ELISA. For supernatant-induced death in ∆M36-infected SVEC4-10 cells, the virus was left on cells for 1 h and the inoculum was removed followed by the addition of supernatants. For imaging, SVEC4-10 cells were plated 5 × 10^5^ in 24-well tissue culture plates. Images were obtained at 20× magnification by IncuCyte Live Cell Imaging Microscopy (Essen Bioscience Inc., Ann Arbor, MI, USA). 

### 2.4. Immunoblot (IB) and Immunoprecipitation (IP)

For extraction of proteins from splenocytes, the spleen was excised from euthanized mice and homogenized as described before [58]. Briefly, spleens were homogenized on ice, centrifuged at 500× *g* at 4 °C for 5 min, pellets resuspended in TAC RBC lysis buffer (Sigma) for 5 min at room temperature in dark (5 mL lysis buffer/spleen), followed by addition of 5 mL warm complete medium. Cells were sedimented, filtered through 100 μM sterile filters, re-sedimented, and dissolved in 1 mL/spleen of ice-cold Triton-X lysis buffer supplemented with protease, as well as phosphatase inhibitors before incubation on ice for 30 min (lysis method details have been described previously [44]). Ultracentrifuged (150,000 rpm at 4 °C for 20 min) lysates were separated from Triton-X insoluble pellet and utilized for immunoblot (IB). Isolation of splenic hematopoietic cells has been described previously [55,59]. Briefly, excised spleens from euthanized mice were injected with 1 mL of murine collagenase III (100 u/mL, Worthington Biochemical Corporation, Lakewood, NJ, USA) in Hank’s balanced salt solution (HBSS) (H9394, Sigma) supplemented with calcium chloride (140 mg/L) or magnesium sulfate (98 mg/L). Spleen was incubated in 1 mL of concentrated collagenase III solution (400 u/mL) for 1 h at 37 °C. Spleens were then forced through a 70 μm Becton Dickenson Cell Strainer using the plunger from a 5 mL syringe, resulting in a filtrate constituting hematopoietic cells (HC). HC were counted and lysed with Triton-X-100 lysis buffer. Infected cells were harvested at 14 hpi and evaluated using a modified protocol from what was described previously [33]. Briefly, 5 × 10^6^ BMDM or SVEC4-10 cells were plated in a 10-cm tissue culture dish. Twenty-four hours post-plating, cells were infected with the virus. Lysates were harvested utilizing 1000 μL of ice-cold Triton-X lysis buffer supplemented with protease and phosphatase inhibitors for 30 min. Ultracentrifuged (150,000 rpm at 4 °C for 20 min) lysates were separated from Triton-X insoluble pellet. Pellets were sonicated for 10 s at 20 watts in 150 μL of disruption buffer (50 mM Tris pH 6.8, 5% β-mercaptoethanol, 2.75% [*w*/*v*] sucrose and 2% [*w*/*v*] SDS). For IP, 800 μL of soluble lysate was incubated overnight by rotation with 30 μL of Protein A/G Agarose Beads (sc-2003, Santa Cruz Biotechnology, Dallas, TX, USA) and 10 μL anti-FADD antibody (sc-6036, clone M-19, Santa Cruz Biotechnology). Prior to start, IP agarose beads were incubated with Triton-X lysis buffer containing 1% bovine serum albumin (BSA) to reduce non-specific binding during IP. Antibodies for immunoblot: rabbit anti-cleaved Casp8 (8592), rabbit anti-cleaved Casp3 (9661), rabbit anti-cFLIP (56343), and rabbit IL-1 β (12242, all from Cell Signaling Technology, Beverly, MA, USA); rat anti-Casp8 (ALX-804-447, Enzo Life Sciences, New York, NY, USA); mouse anti-RIPK1 (610458, BD Biosciences, Beverly, MA, USA); and mouse anti-β-actin (A2228, Sigma-Aldrich, St. Louis, MO, USA).

### 2.5. Statistics

Statistical significance was determined using paired, parametric Student’s t-test for cell culture assays and paired, parametric *t*-test with Welch’s correction for in vivo data. *p*-Values (*p*) of <0.05 were considered significant and indicated with * <0.05, ** <0.01, *** <0.001, **** <0.0001 and n.s. represents non-significant. All statistical analyses were performed using GraphPad Prism 8 (GraphPad Software Inc., La Jolla, CA, USA), and data were graphed using Adobe Illustrator 8 (Adobe Corporation Inc., San Jose, CA, USA).

### 2.6. Data Availability

Further information and requests for resources and reagents should be directed to and will be fulfilled by mocarski@emory.edu and pratyusha.mandal@emory.edu. All raw data are available upon request. This manuscript did not generate new unique reagents. All reagents utilized are described in detail in these methods.

### 2.7. Study Approval

All experiments were conducted with approval (February 4, 2019, Proto 201700351) from the Emory University Biohazard, Chemical Hazard Review and Animal Use (IACUC) Committees.

## 3. Results

### 3.1. vICA Protects Myeloid Cells from MCMV-Induced Apoptosis

**Figure 1:**
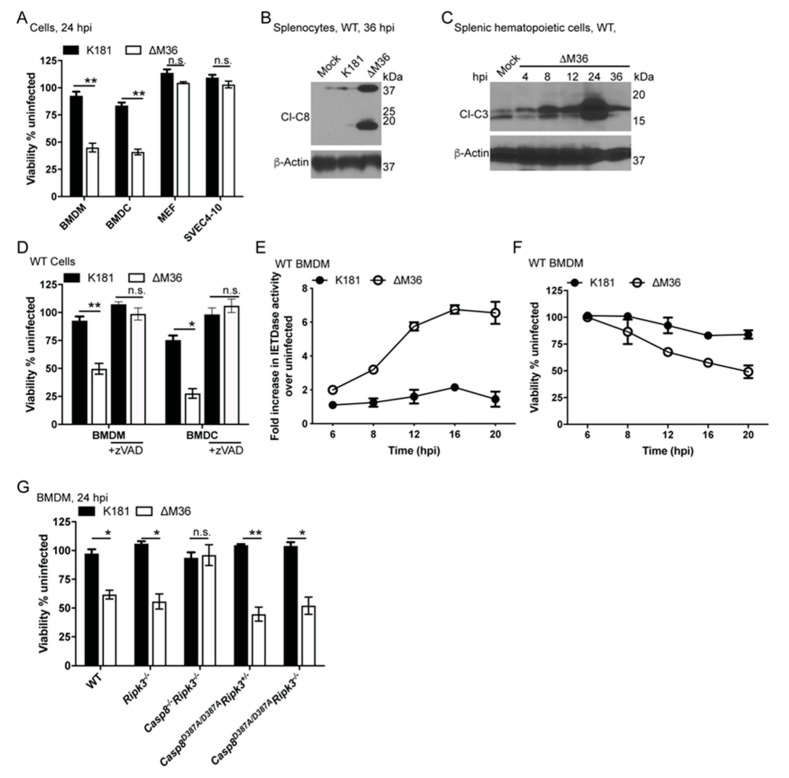
Caspase (CASP)8 activity is required for ∆M36-induced apoptosis. (**A**) Relative viability of bone marrow-derived macrophages (BMDM), bone marrow-derived dendritic cells (BMDC), murine embryonic fibroblasts (MEF), and SVEC4-10 cells infected for 24 h (h) with K181 or ∆M36 at MOI = 10. Relative viability was assessed with respect to parallelly maintained uninfected cells. All viability data are from 2 to 5 independent experiments. (**B**,**C**) Immunoblot (IB) for activated cleavage products of CASP8 (Cl-C8, 18 kDa) (**B**) and CASP3 (**C**) (Cl-C3; 22 and 19 kDa) in splenocytes (**B**) or splenic hematopoietic cells (HC; C) from mice infected with K181 or ∆M36 at indicated hours post-infection (hpi). IB are representatives of data obtained from 10 to 12 mice in 3 to 5 independent experiments for either B or C. (**D**) Relative viability of BMDM and BMDC cells at 24 hpi with K181 or ∆M36 in the absence or presence of zVAD-*fmk* (25 μM). The drug was added 1 hpi with virus and maintained till the end of the assay. E and (**F**) CASP8 activity on substrate sequence isoleucine-glutamine-threonine-aspartic acid (IETD) (**E**) and relative viability (**B**) of infected wild type (WT) BMDM from 6 through 20 hpi. (**G**) Relative viability of BMDM at 24 hpi when indicated genotypes were infected with K181 or ∆M36. Graphs show pooled data from 3 to 5 experiments for each condition. Lines above bars indicate the two groups compared for significance values using paired, parametric Student’s t-test where * is <0.05, ** is <0.01, and n.s. represents nonsignificant.

In order to evaluate the impact of virus-induced apoptosis in different cell types, myeloid and non-myeloid cells were infected with MCMV K181-BAC or deletion mutant ∆M36 (Figure 1A). As expected, [30,31,32,33], BMDM showed susceptibility to ∆M36 virus-induced death. BMDC also showed susceptibility similar to BMDMs, whereas MEFs and endothelial cells (SVEC4-10) showed the expected resistance. Under the conditions used here (MOI of 10), 80% of BMDM and ~100% of MEF or SVEC4-10 cells become productively infected [33]. ∆M36-dependent extrinsic apoptosis limits viral replication in myeloid cells and mice by 24 hpi [11,30,31,32,33]. Inflammatory signaling driven by macrophages (via death or associated cytokine production) is considered the primary contributor to immune cell-mediated clearance and reason for attenuation of ∆M36 in vivo. Splenocytes extracted from homogenized spleens of WT mice intraperitoneally inoculated with K181 expressed viral immediate early (IE)1 protein by 24 hpi when IE1 expression was assayed over 4, 8, 12, 24, and 36 hpi (Figure S1A). By 36 hpi, mutant virus showed compromised replication when spleen homogenates were prepared from WT mice (Figure S1B). vICA interaction with the pro-domain of CASP8 limits the auto-processing of the protease [23]. The absence of vICA during ∆M36 infection allows CASP8 cleavage activation within infected cells [28,29]. Activation of CASP8 (cleaved form p18) was detected in splenocytes of ∆M36-infected WT mice by 36 h post-inoculation (Figure 1B). When CASP3 activation (cleaved forms in p19, p17) was evaluated in the hematopoietic cell compartment mechanically separated from the stromal compartment of individual ∆M36-infected mice over time course from 4 through 36 hpi as described previously [55,59] (Figure 1C), elevated CASP3 cleavage over mock-infected background was evident by 8 hpi and appeared most intense at 24 hpi. The appearance of this virus-induced apoptotic marker (by 8 hpi) was consistent with the known initial virus-induced wave of cytokine production [33,37]. Innate cytokines TNF and IL-1 β were generally elevated in mice infected with K181 virus at this time (Figure S1C).

### 3.2. CASP8 Activation Drives ∆M36-Dependent Apoptosis

To test whether ∆M36-infected BMDM and BMDC died by the expected CASP8-dependent mechanism, we treated both cell types with pan-caspase inhibitor zVAD-*fmk*, starting 1 hpi (Figure 1D). Both WT cell types resisted death when caspase activation was blocked, and BMDM showed a consistent increase in CASP8 activity for substrate isoleucine-glutamine-threonine-aspartic acid (IETD) by 6 to 8 hpi that remained elevated (Figure 1E). By 20 hpi, a majority of cells had lost viability (Figure 1F). *Casp8^−/−^Ripk3^−/−^* cells were protected from this death, whereas *Ripk3^−/−^* cells succumbed (Figure 1G). These data affirm the dispensability of RIPK3 and pro-necroptotic signaling during ∆M36-induced death [33]. The reported [34] impact of HCMV vICA on necroptotic mediator MLKL does not occur during MCMV infection in murine settings, as ∆M36-dependent cell death phenotype is comparable in WT and RIPK3-deficient cells or mice [33]. During TNF-induced signaling, CASP8 activation occurs by autoproteolytic cleavage and processing [60,61]. To assess the requirements for ∆M36-induced CASP8 activation, we infected *Casp8^D387A^* auto-processing mutant cells (Figure 1G) [62]. Despite the resistance of this mutant to conventional stimuli [55,63], ∆M36 infection triggered death in both *Casp8^D387A^Ripk3^+/−^* and *Casp8^D387A^Ripk3^−/−^* cells at levels indistinguishable from WT. Thus, virus-induced apoptotic signaling proceeds independently of CASP8 auto-processing at D387. CASP8 activation and apoptosis occurred within 36 hpi in mice (Figure 1B,C). Consistent with this, replication of ∆M36 was restored to WT levels in spleens from *Casp8^−/−^Ripk3^−/−^* mice (Figure S1B). *Casp8^−/−^Ripk3^−/−^* mice are known to support the dissemination of ∆M36 to the salivary gland by 14 dpi [33] and clodronate-loaded liposome-mediated elimination of phagocytes rescues ∆M36 replication by 48 hpi [11]. In combination, this early rescue of vICA deletion mutant in *Casp8^−/−^Ripk3^−/−^* reinforces prior observations showing that CASP8 is the cognate target of this cell death suppressor. CASP8 is a central innate immune defense mediator against MCMV even though this caspase only makes a modest contribution to an effective antiviral T cell response that brings infection under control [58,64]. Therefore, vICA-mediated inhibition of CASP8 is crucial during the early stages of infection where TNF signaling represents the most prominent inflammatory pathway leading to CASP8 activation [60,61]. *In vivo* production of virus-induced TNF, together with other inflammatory cytokines, correlated with ∆M36-dependent apoptosis (Figure 1B,C and Figure S1C). These data prompted assessment of TNF signaling during CASP8-dependent apoptosis in cultured ∆M36-infected macrophages. 

### 3.3. TNF Production and Signaling Contribute to ∆M36-Induced Apoptosis in Myeloid Cells

**Figure 2:**
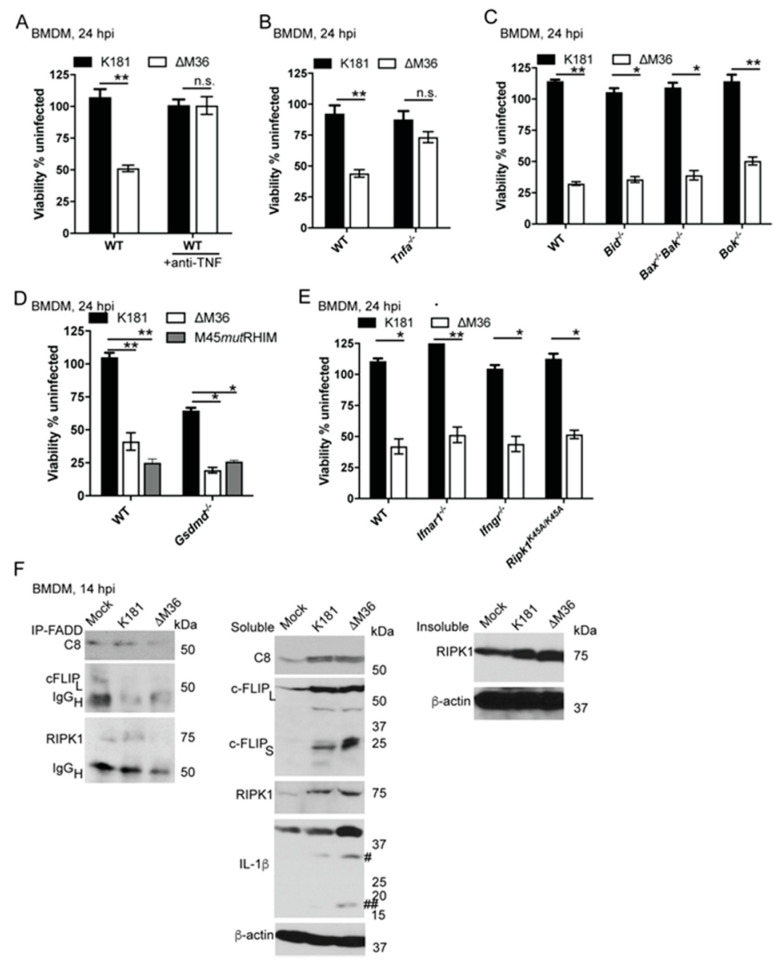
Tumor necrosis factor (TNF) drives ∆M36-dependent apoptosis in BMDM. (**A**) Relative viability of WT BMDM infected with K181 or ∆M36 (MOI = 10) for 24 h in the absence or presence of neutralizing anti-TNF (100 μg/mL). anti-TNF was added in medium 1 hpi. (**B**–**E**) Relative viability of mutant BMDM when compared to WT cells at 24 hpi with indicated viruses. Viability graphs reflect data pooled from 3 to 5 independent experiments each containing 3 replicates. (**F**) IB showing the association of CASP8 (C8; 55 kDa), cellular FLICE inhibitory protein (cFLIP) (full length 55 kDa, indicated as cFLIP_L_) and RIPK1 (75 kDa) with Fas-associated via death domain (FADD) when Triton-X solubilized cell lysates from K181- or ∆M36-infected WT BMDM were subjected to immunoprecipitation at 14 hpi using anti-FADD antibody (left panel). Immunoglobulin (IgG) heavy chain (IgG_H_; 50 kDa) was detected when membranes were incubated with either anti-cFLIP or RIPK1 antibodies. Middle panel indicates total levels of C8, cFLIP_L_, as well as cFLIP short (cFLIP_S_; 25 kDa), RIPK1, and IL-1β with both total (#; 31 kDa) and processed forms (##; 17 kDa) were detected in the soluble cell lysates. Right panel indicates RIPK1 level in the detergent-insoluble fraction. β-actin (38.5 kDa) serves as loading control. IB data are representative of 3 independent experiments. Lines above bars indicate the two groups compared for significance values using paired, parametric Student’s t-test where * is <0.05, ** is <0.001, and n.s. represents nonsignificant.

We compared levels of virus-induced death induced by the parental virus to ∆M36 in WT BMDM when treated with neutralizing anti-TNF sera (Figure 2A), as well as in *Tnfa^−/−^* cells (Figure 2B). Anti-TNF treatment of TNF gene deletion had no impact on K181 infection but dramatically rescued ∆M36-induced apoptosis. The resistance of *Tnfa^−/−^* cells, as well as anti-TNF treated WT cells, demonstrate that TNF is necessary to observe apoptotic signaling (extrinsic apoptosis) in ∆M36-infected macrophages. Thus, TNF activation precedes virus-induced apoptosis. These observations strongly implicate TNFR1, in contrast to observations with pSM3fr-derived M36 mutant virus [31]. Consistent with our observations with K181-derived mutant, macrophage-derived TNF contributes to CD8 T cell-mediated cytotoxicity against ∆M36-infected cells, once MCK2 function has been repaired in pSM3fr-derived virus [11], suggesting some role for vICA beyond the suppression of macrophage apoptosis that we have not sought to address. Importantly, our observations reveal that autocrine TNF signaling drives ∆M36-induced macrophage apoptosis when a fully WT MCMV is employed. We next assessed the contributions from other death signaling pathways to apoptosis, along with any influence of other inflammatory signaling pathways.

### 3.4. Role of Intrinsic (Mitochondrial) Apoptosis, GSDMD, and Type I/II IFNs

Depending on the cell type, CASP8 cleavage of BH3 interacting-domain death agonist (BID) may amplify TNF-dependent death signaling through the release of mitochondrial cytochrome *c*, thereby bridging extrinsic and intrinsic death pathways [60,61]. WT and *Bid^−/−^* cells showed comparable susceptibility to ∆M36-induced apoptosis, indicating BID does not contribute to death signaling in these myeloid cells (Figure 2C). Furthermore, BMDM lacking the critical contributors to intrinsic apoptosis, Bcl-2 associated X protein (BAX) and Bcl-2 homologous antagonist killer (BAK) proteins necessary for the mitochondrial pathway, or Bcl-2 related ovarian killer (BOK) were all susceptible to ∆M36-induced death. Notably, ∆M36 retains virus-encoded suppressors of mitochondrial cell death signaling [12,24,28]. Although the mitochondrial pathway would be blocked by these suppressors, these data formally establish that prominent Bcl2 family members, BID, BAX, BAK, and BOK do not contribute to ∆M36-induced death of macrophages. GSDMD executes pyroptotic death [65] and has been reported to enhance apoptosis [66]. In certain settings such as during *Yersinia* infection of macrophages, CASP8 is able to substitute for pro-pyroptotic CASP1 and CASP11 to cleave GSDMD and execute death [67]. We investigated whether GSDMD contributed to ∆M36-induced CASP8 signaling. *Gsdmd^−/−^* cells were just as susceptible to ∆M36-induced death as WT cells (Figure 2D). *Gsdmd^−/−^* cells also were susceptible to M45*mut*RHIM-induced necroptosis. Thus, in contrast to other inflammatory settings where pyroptosis and apoptosis signaling may be interlinked with necroptosis [68], GSDMD plays no role in CMV-induced death pathways. These observations are consistent with our earlier report [33]. IFNs synergize with TNF to disrupt development or to mediate tissue damage during endotoxemia, hepatitis, as well as viral infections [55,56,69,70]. Type I IFN appears within 4 h [59] and type II IFN appears within 18 h in spleens during MCMV infection [33]. To assess autocrine contributions from IFNα/β or IFNγ during ∆M36-induced apoptosis, we infected WT, *Ifnar1^−/−^*, and *Ifngr^−/−^* BMDM (Figure 2E). ∆M36 infection triggers cell death in all genotypes, indicating IFN-signaling does not contribute to this TNF-dependent pathway. In contrast, *Ifnar1^−/−^* mice showed higher levels of WT or ∆M36 replication in spleen at 36 hpi (Figure S1A). As expected, type I IFN limited viral replication such that parental virus levels were elevated at least 100-fold in *Ifnar1^−/−^* mice compared to WT mice. These mutant mice were highly susceptible to WT virus when spleen, liver, and lung were assessed at 3 dpi; however, they resisted ∆M36 replication (Figure S1D). ∆M36 showed similar, poor dissemination to the salivary gland in either WT or *Ifnar1^−/−^* mice (Figure S1E). These observations show type I and II IFN signaling are dispensable for ∆M36-dependent macrophage apoptosis as well as observed compromised dissemination. Overall, ∆M36-induced apoptosis appears to proceed by direct CASP8-mediated cleavage of executioner caspases without a contribution of other prominent inflammatory signaling pathways. 

### 3.5. Role of FADD-Associated Extrinsic Death Complex

CASP8 activation is known to require the formation of a FADD-dependent extrinsic death complex (association of FADD-CASP8-cFLIP_L_-RIPK1-RIPK3), whether signaling is initiated via TNFR1 or RIPK1/RIPK3 [5,30,44]. Aggregated receptor-interacting protein homotypic interaction motif (RHIM)-dependent RIPK1/RIPK3 complexes are known to accumulate in the insoluble fraction of cell lysates in all settings where they form [44,60]. Surprisingly, when Triton-X solubilized protein lysates from WT BMDM mock-treated or infected with either virus were immunoprecipitated with anti-FADD sera, ∆M36 infection (14 hpi) had no impact on the basal FADD-CASP8 complex detected in mock-treated cells (Figure 2F left panel), a result reminiscent of evaluations performed at 12 hpi [33]. FADD-associated RIPK1 (75 kDa) appeared slightly enhanced over mock in K181 but not in ∆M36-infected cells. It should be noted that variation in the IgG heavy chain (p50) represents differences in protein loaded on the gel that did not obscure the observation. Neither virus infection exhibited any association of FADD with full-length (55kDa) cFLIP (also known as cFLIP_Long_ or cFLIP_L_). Thus, TNF-dependent CASP8 activation in ∆M36-infected cells does not result in a distinguishable complex IIb or ripoptosome, in stark contrast to other extrinsic apoptotic settings [4,44,71]. Importantly, these observations align with our observations on herpes simplex virus (HSV)1 infection where infection suppresses TNFR1-dependent RIPK1/RIPK3/CASP8 aggregates [72]. MCMV ∆M36 infection may behave similarly owing to RHIM suppression by M45, which is retained in this mutant. When Triton-X-soluble fractions were evaluated, levels of unprocessed CASP8 (p55), cFLIP_L_ (p55), and RIPK1 (p75) all increased during infection with either virus (Figure 2F, middle panel). These observations suggest that RIPK1, CASP8, and cFLIP_L_ all contribute to the cell autonomous signaling network associated with pathogen sensing such that infection induces their expression, thereby priming cells to die unless suppressed by vICA. When the insoluble fraction was examined, RIPK1 was elevated following infection with either virus. ∆M36-infected cells exhibited the highest levels of accumulated RIPK1 (Figure 2F, right panel). Therefore, ∆M36-dependent CASP8 activation does not enhance a complex IIb but proceeds, owing to enhanced levels of pro-apoptotic proteins. Infection (either with K181 or ∆M36) also triggered both isoforms of cFLIP (cFLIP_Short_ or cFLIP_S_) (Figure 2F, middle panel). cFLIP_L_ closely resembles full-length procaspase 8, whereas splice variant cFLIP_S_ (p25) is truncated, composed primarily of two tandem repeats of the death effector domain (DED) [73]. Both isoforms of cFLIP contribute to the regulation of extrinsic apoptosis [74]. Thus, MCMV-infected BMDM induce cFLIP splicing. CASP8 is a recognized contributor to cytokine signaling in infected or stressed cells [33,75,76,77]. Of particular significance is the processing and activation of the inflammatory cytokine IL-1 β via the NLRP3-inflammasome, where CASP8 acts in addition to CASP1 as an effector and regulator [33,75,76,77,78]. Where full-length inactive IL-1β (31 kDa) was induced by K181 infection over mock, ∆M36 infection exhibited increased cytosolic levels of both full-length and processed (activated) IL-1 β (17 kDa), suggesting that ∆M36-induced apoptosis is accompanied by enhanced inflammation. These observations directly challenge the notion that apoptosis (extrinsic or intrinsic) is inherently anti-inflammatory [3] by demonstrating that MCMV-triggered extrinsic apoptosis proceeds in close association with inflammatory signaling. Thus, during infection, vICA mitigates CASP8-driven macrophage death as well as inflammatory signaling.

### 3.6. TNF Mediates Death in Non-Myeloid Cells

**Figure 3:**
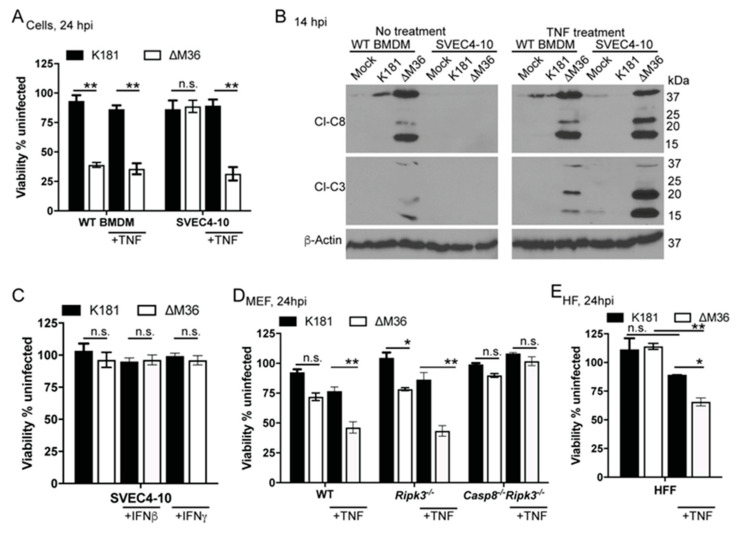
TNF drives ∆M36-dependent apoptosis in endothelial cells and fibroblasts. (**A**). Relative viability of WT BMDM and SVEC4-10 cells infected with K181 or ∆M36 (MOI = 10) for 24 h in the absence or presence of murine TNF (25 ng/mL). TNF was added to medium 1 hpi. Graph reflects data pooled from three independent experiments, each containing 3 replicates. (**B**). IB showing the appearance of Cl-C8 and Cl-C3 with loading control β-actin 14 hpi in WT or SVEC4-10 cells under indicated conditions. Data are representative of 2 independent experiments. (**C**–**E**). Relative viability of SVEC4-10 treated with no cytokine, murine IFNβ or IFNγ (100 ng/mL each), (**C**), MEFs of indicated genotypes treated with murine TNF (25 ng/mL) (**D**) or HF treated with human TNF (100 ng/mL) (**E**) and infected with K181 or ∆M36 (MOI = 10) at 24 hpi. All cytokines were added to the medium at 1 hpi with virus. C-E reflect data pooled from two independent experiments, each containing 3 replicates. Lines above bars indicate the two groups compared for significance values using paired, parametric Student’s *t*-test where * is <0.05, ** is <0.01, and n.s. represents nonsignificant.

Myeloid cells are recognized as primary producers of innate cytokines during inflammatory settings [55,79] that restrict pathogen replication by amplifying innate inflammatory pathways including cell death in responding myeloid or non-myeloid cells [55,80,81]. vICA-deficient HCMV or MCMV infects and replicates in fibroblasts without inducing death [30,33,44]. However, none of these cell culture experiments were performed in presence of TNF or other cytokines likely to influence outcomes during natural infection of tissues. To test whether ∆M36 infection would collaborate with TNF to induce the death of non-myeloid cells, we treated apoptosis-resistant endothelial cells with TNF, IFNβ, or IFNγ starting 1 hpi (Figure 3A–C and Figure S2A). Only TNF supported ∆M36-induced death of SVEC4-10 cells. After TNF addition, ∆M36-infected SVEC4-10 cells exhibited activation of both CASP8 and CASP3 (Figure 3B) as well as distinct apoptotic morphology (Figure S2A). The addition of neutralizing anti-TNF sera to cell supernatants blocked death induction (Figure S2B). Notably, the addition of TNF did not alter (i) K181-dependent death in either BMDM or SVEC4-10, or (ii) ∆M36-dependent apoptosis in BMDM (Figure 3B), suggesting that the TNF autocrine signaling loop cannot be further enhanced in ∆M36-infected BMDM by exogenous TNF. In inflammatory settings, TNFR1-dependent extrinsic apoptosis is known to require kinase activity from RIPK1 [82]. The specific small-molecule inhibitor of RIPK1 kinase activity (GSK’963; [56]) failed to prevent TNF-mediated death in ∆M36-infected SVEC4-10 cells, demonstrating dispensability of RIPK1 kinase activity, an observation consistent with the results in BMDM [33]. To address whether TNF drove apoptosis in other ∆M36-susceptible, death resistant cell types, we treated ∆M36-infected MEFs or HFs with TNF (Figure 3D,E). Despite being a mouse virus, K181 enters HFs and express early gene products [8]. TNF-mediated death in infected WT MEFs (Figure 3D) or HF (Figure 3E) confirming that signaling by this cytokine drives ∆M36-induced death in murine (endothelial cells, as well as fibroblasts) or human (fibroblast) cells. *Ripk3^−/−^* MEFs succumbed but *Casp8^−/−^Ripk3^−/−^* cells completely resisted this apoptosis, establishing the requirement for CASP8 during ∆M36-induced death in permissive non-myeloid cell types. ∆M36-induced death in human fibroblasts indicated that the MCMV M36-encoded vICA acts in a species-independent fashion, similar to what we found in studies of M45mutRHIM-induced necroptosis [8]. Although not evaluated here, HCMV UL36 is known to rescue cells from ∆M36 deficiency in murine settings [53]. These observations suggest future studies focused on fully characterizing HCMV vICA function are necessary to reveal distinctions between UL36 and M36 beyond CASP8 inhibition, especially given the reported function of UL36 to stabilize MLKL [34]. Importantly, we confirm an evolutionary preserved function of vICA as the suppressor of TNF-dependent extrinsic death in infected cells across species.

### 3.7. Myeloid Cells Produce TNF

**Figure 4:**
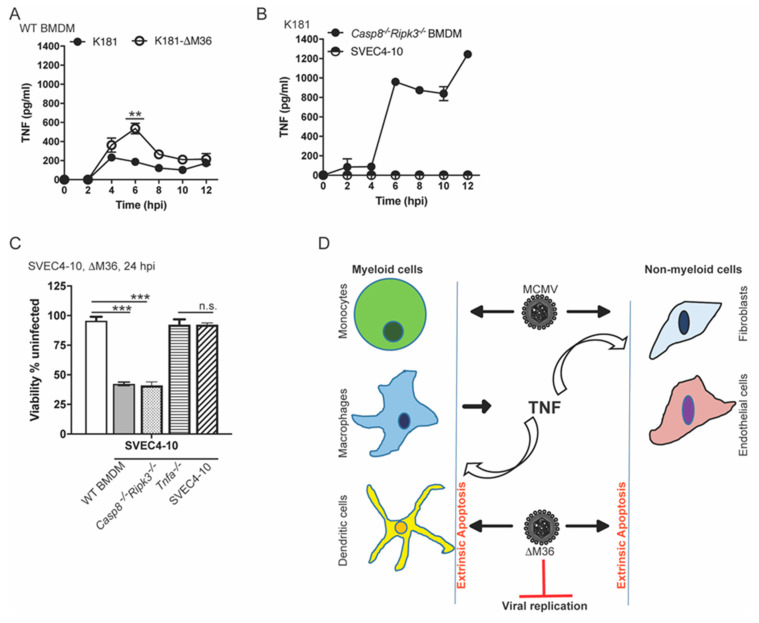
Macrophage-derived TNF drives ∆M36-dependent apoptosis in endothelial cells. (**A**,**B**). TNF quantities in supernatant collected from K181- or ∆M36-infected (MOI = 10) WT BMDM (**A**) and *Casp8^−/−^Ripk3^−/−^* BMDM or SVEC4-10 cells infected with K181 (MOI = 10) (**B**) over the indicated time course. Graphs reflect data pooled from one ELISA experiment analyzing data from 3 independent infection experiments each containing 2 replicates for all conditions. (**C**). Relative viability of untreated SVEC4-10 cells infected with ∆M36 (MOI = 10) for 24 h and in absence or presence of supernatants collected at 6 hpi from ∆M36-infected BMDM (WT, *Casp8^−/−^Ripk3^−/−^* or *Tnfa^−/−^*) or SVEC4-10 cells. Untreated SVEC4-10 cells were infected for 1 h and then media or supernatants were added. Data are from one experiment with 3 replicates. (**D**). Model for MCMV and vICA mutant infection of myeloid (monocytes, macrophages, or dendritic) cells and non-myeloid (fibroblasts or endothelial cells). TNF is produced by myeloid cells that kill myeloid cells in an autocrine loop and non-myeloid cells in a paracrine loop. Lines above bars indicate the two groups compared for significance values using paired Student’s t-test where ** is <0.01, *** is < 0.001, and n.s. represents nonsignificant.

We next sought to establish whether macrophage-derived TNF suffices to drive the death of ∆M36-infected non-myeloid cells. BMDM produced but SVEC4-10 cells did not produce TNF during infection (Figure 4A,B). K181 or ∆M36-infected WT BMDM produced TNF from 4 through 12 hpi (Figure 4A). TNF was not detected in the supernatant from infected SVEC4-10 cells over this time course (Figure 4B). TNF levels were significantly higher in supernatants from ∆M36-infected cells at 6 hpi, indicating that vICA suppresses death-associated inflammation during infection of myeloid cells. Taken together with ∆M36-induced IL-1 β processing at 14 hpi (Figure 2F), these observations bring to light an unexpected link between proapoptotic and inflammatory cytokine signaling. CASP8 was dispensable for TNF production by macrophages such that K181-infected *Casp8^−/−^Ripk3^−/−^* BMDM produced detectable TNF, at levels higher than WT cells (Figure 3B). A possible contribution from RIPK3 and/or CASP8 in restraining K181-triggered TNF production from BMDM cannot be ruled out. Importantly, CASP8-independent production of TNF drives autocrine TNF-dependent CASP8 activation in vICA deficient mutant-infected macrophages.

### 3.8. Paracrine TNF Signaling Drives CASP8-Dependent Apoptosis in Non-Myeloid Cells

We next determined whether BMDM-derived paracrine TNF signaling determined death outcome in endothelial cells. Cell-free supernatants collected from ∆M36-infected WT, *Casp8^−/−^Ripk3^−/−^*, and *Tnfa^−/−^* BMDM, as well as SVEC4-10 cells, at 6 hpi, and applied to ∆M36-infected, death-resistant SVEC4-10 cells for 24 h (Figure 4C). WT and *Casp8^−/−^Ripk3^−/−^* supernatants killed infected SVEC4-10 cells but *Tnfa^−/−^* conditioned medium failed to kill these cells, identifying TNF necessary for death. Supernatants from SVEC4-10 cells did not induce death, consistent with a lack of TNF production. Thus, during ∆M36 infection, both myeloid and non-myeloid cells die by the collaboration of TNF-dependent and virus-induced CASP8-mediated apoptotic signaling. Myeloid cells produce the necessary proinflammatory cytokine necessary to observe virus-induced death in either cell type. Although prior studies with fully WT MCMV have included mice to investigate collaboration between TNF and virus-infected, they did not recognize the critical link between inflammation and death likely because of the results from cultured cells [30,33,44] without employing TNF or supernatants from infected myeloid cells. Only through the objective assessment of appropriate mutant cells and animals did these critical features of vICA biology emerge.

Here, we establish TNF as the primary mediator of CASP8-dependent apoptosis in CMV-infected myeloid cells and non-myeloid cells. Infected myeloid cells produce TNF (along with other cytokines); TNF mediates autocrine and paracrine death signaling in myeloid and non-myeloid cells, respectively (Figure 4D). Death in all TNF-responsive cells eliminates ∆M36-infected cells and limits the replication fitness of this virus in the host. Thus, normalization of ∆M36 replication in *Casp8^−/−^Ripk3^−/−^* mice (Figure S1A), as first revealed in Daley-Bauer et al. [33], involves the rescue of mutant virus replication in myeloid as well as non-myeloid cell types from extrinsic apoptosis. *In vivo,* cell death is unlikely to be limited to macrophages, monocytes, and other myeloid cells. CASP8, as well as cFLIP_L_ and RIPK1, emerge to be central cell autonomous innate pathogen sensors such that levels of all these proteins increase during infection. This signaling primes infected cells for TNF-dependent elimination, which occurs through apoptosis or necroptosis unless blocked by specific virus-encoded suppressors. 

## 4. Discussion

Our assessment of MCMV-infected myeloid or non-myeloid cells reinforces evidence that vICA suppression of CASP8 activation and apoptosis acts against TNF-dependent signaling during infection. Functional conservation between MCMV and HCMV ensures a vICA-mediated block of virus-induced death in either host. It is likely that a similar interplay between this inflammatory cytokine and infection controls outcome during HCMV infections. These observations show that the pathway leading to apoptosis during vICA-deficient virus infection relies on TNF-dependent activation of CASP8 in myeloid or non-myeloid cells. Thus, vICA protects from cell death and promotes viral replication in a broader range of cell types than previously recognized. Our observations on TNF remains relevant to HCMV infection and associated pathologies [17,83]. Implications of these data extend to the reported ability of HCMV-encoded vICA to stabilize MLKL [34]. We believe this potentially results from the inhibition of the necrosome complex (TNFR1-dependent association of RIP kinases in presence of CASP8 inhibition) as observed during HSV-1 infection [72]. During natural infection, innate cytokines are produced in tissues, and vICA-suppression axis is necessary to prevent the elimination of infected cells. In absence of vICA, cells undergo CASP8-dependent death, a phenomenon that had been overlooked for non-myeloid populations in prior cell culture studies [30,33]. This is due to the fact that macrophages produce TNF and hence death is observed in this cell type due to autocrine cytokine signaling. We predict the normalization of vICA deletion mutant virus in CASP8-deficient hosts results from a combined rescue of death in any TNF-responsive cell type regardless of whether myeloid or non-myeloid. Assessment of the contribution from TNF (and other innate cytokines) to other programmed cell pathways, including associated with CMV infection including intrinsic apoptosis and necroptosis, is necessary to fully resolve the host–pathogen interaction profile for this virus. Additionally, a direct comparison between fully WT MCMV (containing the full-length MCK2), and previously utilized pSM3fr (MCK2-deficient), as well as its marker rescue pSM3fr-repair [45], must be performed to discern additional differences that exist in available ∆M36 mutant virus strains.

The signaling through which TNF drives CASP8-activation and apoptosis in vICA deletion mutant virus-induced macrophages appears distinct from the other settings of extrinsic apoptosis [44,60,71,84,85]. Either K181 or ∆M36 drives elevated levels of full-length CASP8, cFLIP, and RIPK1 proteins, while the FADD-CASP8-RIPK1-cFLIP_L_ complex remains similar to mock-infected cells. These observations directly contrast the existing notion that an enhanced FADD-CASP8 is necessary to promote CASP8 processing and raises a few possibilities that require investigation [44,60,71,84,85]. Fibroblasts, endothelial cells, and other non-myeloid cells that have been the focus of TNF-driven death studies form detectable complex IIb or ripoptosome [44,60,71,84,85,86]. Even though macrophages remain susceptible to TNF signaling-dependent death, the formation of a similar complex IIb is not readily detected in these cells during the execution of extrinsic death pathways [87]. Our observations expose that the basal FADD-CASP8 is sufficient to collaborate with the infection-driven elevation in levels of CASP8, cFLIP, and RIPK1 for unleashing extrinsic apoptosis when vICA is absent. Our study highlights a distinct TNF response and extrinsic apoptotic signaling exists in different cell types, a finding that is likely to influence inflammatory signaling during other viral infections or inflammatory diseases. The lack of elevated FADD-complex formation in MCMV-infected cells may also result from alternate MCMV suppression strategies in line with observations with HSV1 [72]. MCMV inhibits this aggregation independently of vICA, possibly in collaboration with the RHIM-signaling suppressor M45. In agreement with this, ∆M36/M45*mut*RHIM double mutant virus exhibits an enhanced association between FADD, CASP8, and RIPK1 at 12 hpi [33]. Importantly, CASP8 plays a central pathogen sensing role that primes cells for apoptosis and collaborates with the TNF-signaling for the execution when vICA is absent during infection [31]. In combination with previous observations that vICA stabilizes the necroptotic executioner MLKL in human cells [34], our data indicate vICA-dependent subversion strategy is likely to influence necroptosis beyond extrinsic apoptosis. It is important to note that GSDMD does not contribute to ∆M36-induced apoptosis or M45*mut*RHIM-dependent necroptosis. These observations, along with the absence of GSDMD activation in K181-infected WT BMDM [33], imply that MCMV may encode a yet-to-be recognized inhibitor of GSDMD activation and pyroptosis. The enhancement of cFLIP_S_ during ∆M36 infection also reveals the impact of vICA on cFLIP processing. Mammalian cell fate and induction of extrinsic apoptosis rely on comparative levels of cFLIP isoform expression [73], highlighting the need to assess contributions from this apoptotic protein during CMV infection. At this point, it is not possible to say whether vICA directly binds cFLIP pro-domains or whether cFLIP regulation occurs indirectly via suppression of CASP8 activation. The induction of death in ∆M36-infected HF also raises the possibility that CASP10 and CASP8 are both targets of vICA in human cells [88].

Our study elaborates on the suggested impact of vICA directly on inflammatory signaling in infected macrophages along with cell death [31]. Our data do not distinguish whether vICA-dependent suppression of cytokine signaling (IL-1β processing or TNF production) is associated with suppression of apoptosis or is independent of death signaling. It is well recognized that CASP8 scaffold promotes death-independent inflammation downstream to DR or toll-like receptors [76,89]. Any death-independent pro-inflammatory function of CASP8 during CMV infection remains to be evaluated, even though our observations implicate this protease in restriction of MCMV-triggered innate and adaptive immune cell numbers [90]. vICA binding to the CASP8 pro-domain likely suppresses both death and inflammatory scaffold function. CASP8 scaffold-dependent inflammatory functions acting independently of its protease unleashes RIPK3-dependent necroptotic signaling that underlies the embryonic lethality of *Casp8^−/−^* mice [60,91]. Given the identified anti-inflammatory function of vICA, it will be worthwhile to assess whether vICA expression rescues the embryonic lethality or other CASP8-dependent inflammatory pathologies such as bacterial pneumonia and endotoxemia [55]. Overall, it seems that in infected hosts, vICA promotes viral replication by suppressing TNF signaling-dependent CASP8-mediated cell death, as well as associated CASP8-dependent inflammatory signaling. On this note, type I IFN signaling was not required for macrophage apoptosis (Figure 2) or virus dissemination (Figure S1), but *Ifnar1^−/−^* normalized replication of K181 and ∆M36 replication at 36 hpi suggesting that in mammals macrophage death-independent innate inflammatory signaling may not be the sole contributor to clearance of ∆M36 within 36 hpi. Further focused investigations will identify how does the IFN signaling interfaces with the viral death-suppression mechanism. The relevance of innate cytokines collaborating with virus-induced death, as well as suppression of death, needs careful assessment in different cell types within the host during CMV-infection. 

In conclusion, we show that during MCMV infection, myeloid cells produce TNF that mediates autocrine activation of CASP8 and cross-talks with non-myeloid cells for this extrinsic signaling. vICA suppresses this CASP8-dependent cell death in myeloid cells, endothelial cells, and fibroblasts and subverts inflammatory death signaling triggered by TNF. Thus, myeloid cells (primary targets for CMV infection) are the producers, amplifiers, as well as responders to TNF, while non-myeloid cells function primarily as responders in this signaling cascade. CMV replication and infection require a complex interplay between an innate cytokine (TNF), cell autonomous mediator of extrinsic death (CASP8), and vICA-dependent subversion of the host inflammatory signaling.

## Supplementary Materials

The following are available online at https://www.mdpi.com/1999-4915/12/11/1221/s1, Figure S1: Role of CASP8 and type I IFN signaling during MCMV replication in vivo. Figure S2: TNF-induced apoptosis in ∆M36-infected endothelial cells.
