# Supplementary material for: TNF Signaling Dictates Myeloid and Non-Myeloid Cell Crosstalk to Execute MCMV-Induced Extrinsic Apoptosis
Source: Viruses. 2020 Oct 28;12(11):1221. doi: 10.3390/v12111221 (PMC7693317; doi:10.3390/v12111221)
Supplement: Supplementary file 1 [file viruses-12-01221-s001.pdf]

Supplemental Figures

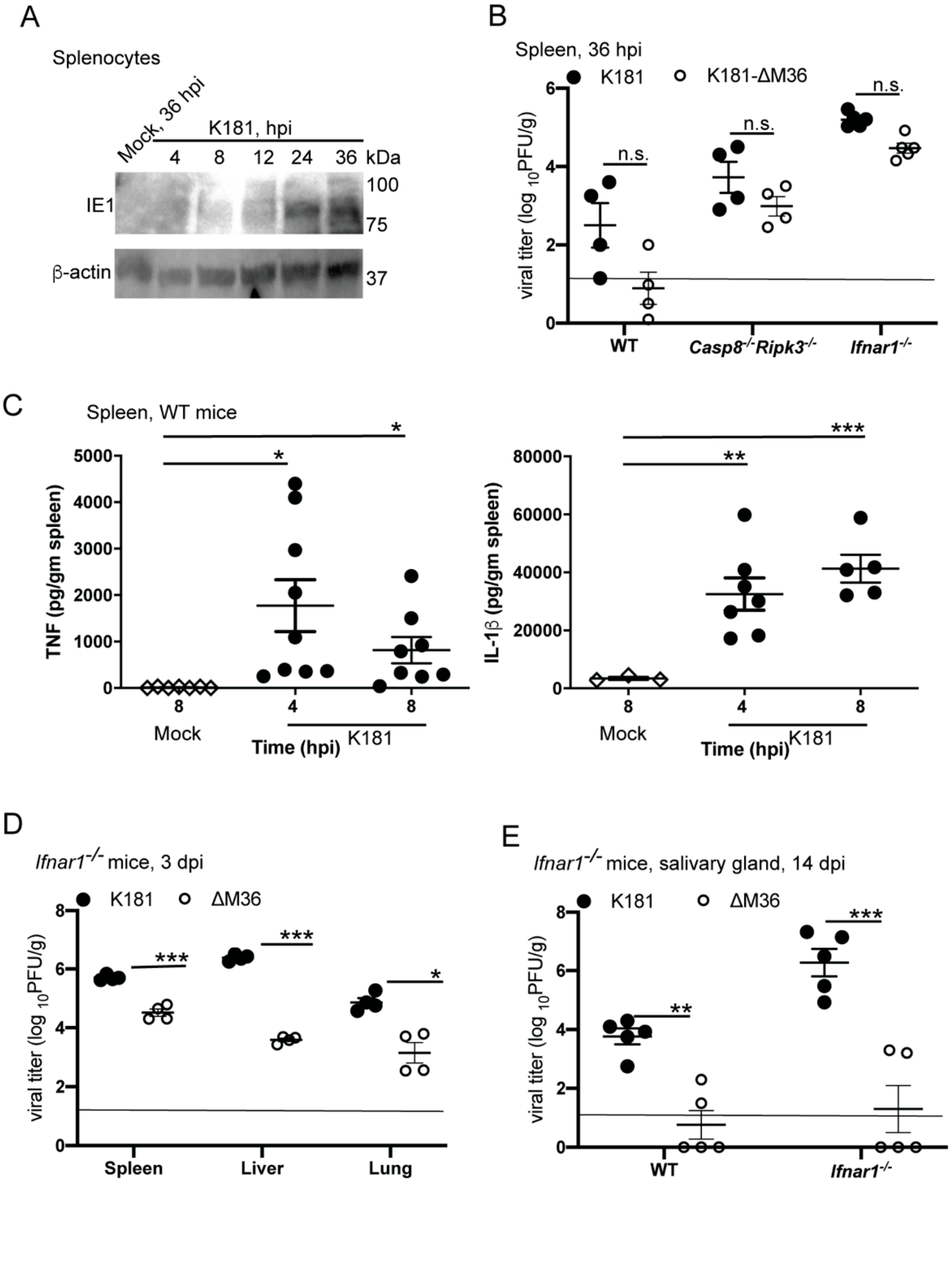

Figure S1. Role of CASP8 and type I IFN signaling during MCMV replication *in vivo*. A. IB blot to determine IE1 (89 kDa) expression levels with β-actin (38.5 kDa) loading control in splenocyte protein lysates extracted from spleens of WT mice inoculated intratracheally with 10^6 PFU K181 at indicated times post infection. Each lane represents lysate from a single mouse. Mock splenocytes were harvested form media-inoculated mouse at 36 hpi. B. Viral titer in spleens from WT, *Casp8*$^{-/-}$*Ripk3*$^{-/-}$ or *Ifnar1*$^{-/-}$ mice at 36 hpi with indicated viruses. All *in vivo* infections were performed as described for IB of IE1 (A). Each data point represents one mouse. Line inside graph indicates limit of detection for spleen (~18 PFU/g). C. TNF and IL-1β quantities in splenic lysate from WT mice infected with K181 at 4 or 8 hpi. Data are from two independent experiments. D and E. Viral titer in indicated organs from *Ifnar1*$^{-/-}$ at 3 dpi (C) or 14 dpi (D). Line indicates limit of detection for each organ for indicated time points (~20 PFU/g at either 3 or 14 dpi). Data is from 1 experiment for each group. Lines top of data points indicate statistical comparison by paired t test using Welch's correction between groups such that *is <0.05, **is < 0.01, ***$<.001$ and n.s. represents non significant.

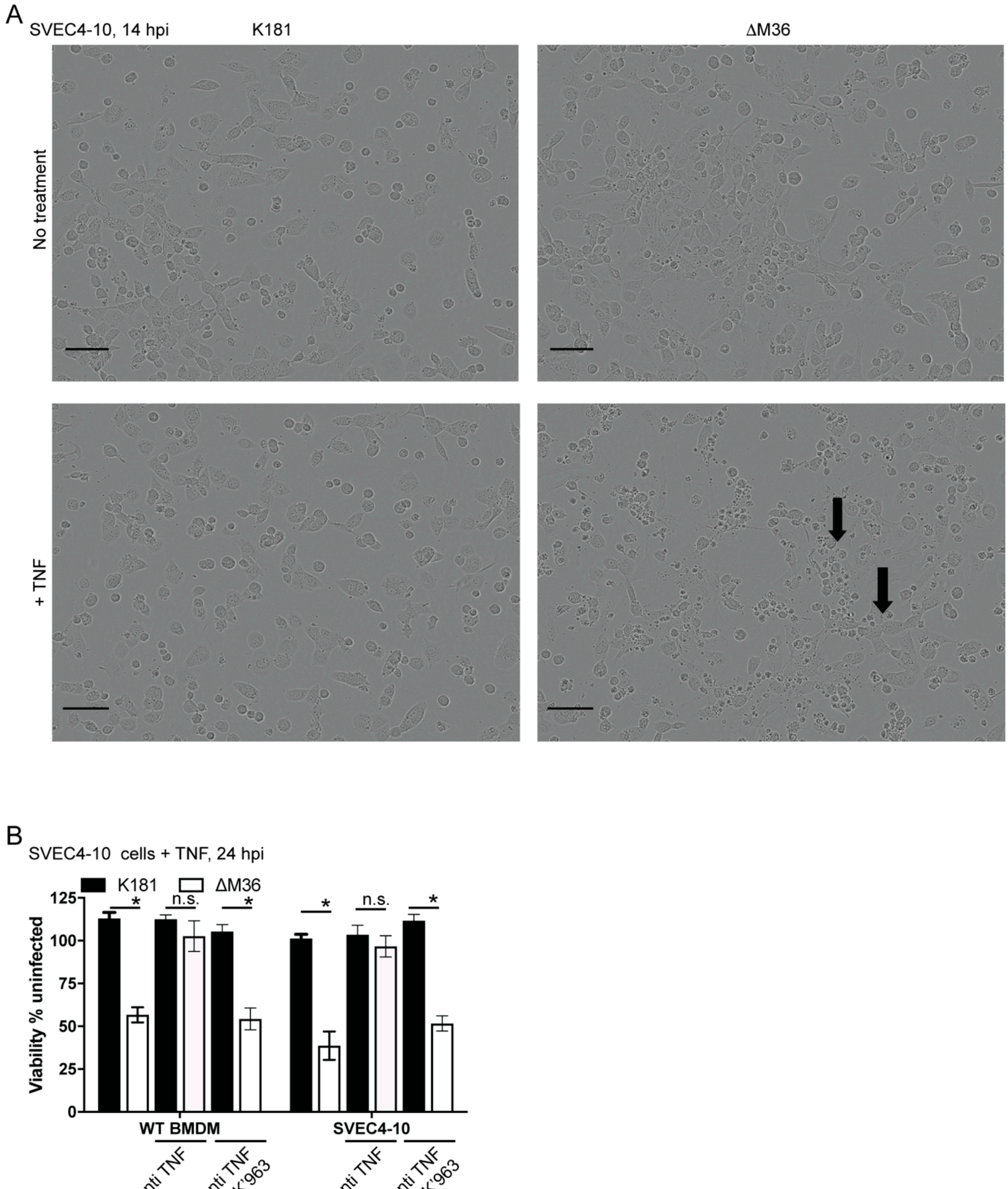

Figure S2. TNF-induced apoptosis in ΔM36-infected endothelial cells. A. Images of K181-or ΔM36-infected SVEC4-10 cells in absence or presence of TNF. Arrows indicate appearance of apoptotic morphology and bars indicates 200 μM resolution where images were captured by IncuCyte. Pictures are representative from two independent experiments each containing two replicates for all conditions. B. Relative viability of ΔM36-infected, TNF-treated WT BMDM and SVEC4-10 cells, in presence of neutralizing anti-TNF (100 ng/ml) or GSK'963 (5 μM). Graphs show pooled data from one experiment for each condition with 3 replicates. Lines above bars indicated the two groups compared for significance values using paired, parametric Student's t-test where * is < 0.05, and n.s. represents non-significant.
